# Measurement of Polybrominated Diphenyl Ethers and Metabolites in Mouse Plasma after Exposure to a Commercial Pentabromodiphenyl Ether Mixture

**DOI:** 10.1289/ehp.10011

**Published:** 2007-04-06

**Authors:** Xinghua Qiu, Minerva Mercado-Feliciano, Robert M. Bigsby, Ronald A. Hites

**Affiliations:** 1 School of Public and Environmental Affairs, Indiana University, Bloomington, Indiana, USA; 2 Department of Pharmacology and Toxicology, School of Medicine, Indiana University, Bloomington, Indiana, USA; 3 Department of Obstetrics and Gynecology, School of Medicine, Indiana University, Indianapolis, Indiana, USA

**Keywords:** BDE-153, bromophenols, DE-71, hydroxylated PBDEs, hydroxylation, polybrominated diphenyl ethers

## Abstract

**Background:**

Previous studies have shown that polybrominated diphenyl ethers (PBDEs) behave as weak estrogens in animal and cell culture bioassays. *In vivo* metabolites of PBDEs are suspected to cause these effects.

**Objectives:**

To identify candidate metabolites, mouse plasma samples were collected after continuous oral and subcutaneous exposure to DE-71, a widely used commercial pentabromodiphenyl ether product, for 34 days.

**Methods:**

Samples were extracted, separated into neutral and phenolic fractions, and analyzed by gas chromatographic mass spectrometry.

**Results:**

In the plasma samples of orally treated animals, 2,2′,4,4′,5,5′-hexabromodiphenyl ether (BDE-153) represented 52% of total measurable PBDEs, whereas it represented only 4.3% in the DE-71 mixture. This suggested that BDE-153 was more persistent than other congeners in mice. Several metabolites were detected and quantitated: 2,4-dibromophenol, 2,4,5-tribromophenol, and six hydroxylated PBDEs. The presence of the two phenols suggested cleavage of the ether bond of 2,2′,4,4′-tetrabromodiphenyl ether (BDE-47) and 2,2′,4,4′,5-pentabromodiphenyl ether (BDE-99), respectively. The hydroxylated (HO)-PBDEs might come from hydroxylation or debromination/hydroxylation. Among the quantitated hydroxylated metabolites, the most abundant was 4-HO-2,2′,3,4′-tetra-BDE, which suggested that there was a bromine shift during the hydroxylation process. *para-*HO-PBDEs have been proposed to behave as endocrine disruptors.

**Conclusions:**

There seem to be three metabolic pathways: cleavage of the diphenyl ether bond, hydroxylation, and debromination/hydroxylation. The cleavage of the diphenyl ether bond formed bromophenols, and the other two pathways formed hydroxylated PBDEs, of which *para-*HO-PBDEs are most likely formed from BDE-47. These metabolites may be the most thyroxine-like and/or estrogen-like congeners among the HO-PBDEs.

Polybrominated diphenyl ethers (PBDEs) are ubiquitous environmental contaminants that are found in both abiotic and biotic environmental samples (Hites 2004; Streets et al. 2006). PBDEs are used as flame retardants; the three main commercial types of PBDE are penta-BDE, octa-BDE, and deca-BDE. DE-71, a widely used commercial penta-BDE product, is generally composed of 50–60% penta-BDE congeners, 24–38% tetra-BDE congeners, and 4–8% hexa-BDE congeners (Birnbaum and Staskal 2003). Since the 1970s, penta-BDE has been used as a flame retardant in polyurethane foam–containing consumer goods such as carpet padding, sofas, and mattresses; this flame retardant can account for up to 30% by weight of the foam (Hale et al. 2002). DE-71 also has minor uses in phenolic resins, polyesters, and epoxy. Despite its relatively small global production and usage compared with deca-BDE, the congeners in penta-BDE, such as 2,2′,4,4′-tetra-bromodiphenyl ether (BDE-47), 2,2′,4,4′,5-pentabromodiphenyl ether (BDE-99), and 2,2′,4,4′,5,5′-hexabromodiphenyl ether (BDE-153), are the most common PBDE congeners found in environmental samples, especially in biotic samples (Hites 2004), suggesting that these congeners are persistent and bioaccumulative.

Although the toxicity of PBDEs is not well understood, these compounds may be neurotoxic, thyrotoxic, estrogenic, and carcinogenic (Siddiqi et al. 2003). Their toxicity might be due to hydroxylated PBDE metabolites (HO-PBDEs) formed in animals. Although HO-PBDEs are less persistent than PBDEs, they may have more severe biological effects. For example, HO-PBDEs have thyroxine-like and estrogen-like chemical structures (Meerts et al. 2001), and thyroid hormone–like HO-PBDE congeners have been shown to bind competitively with human transthyretin (TTR), a transport protein for the thyroid hormones, thyroxine and thyronine (Meerts et al. 2000). The competitive binding of HO-PBDE to TTR provides a mechanism that can potentially result in disrupted thyroxine homeostasis. Putative PBDE metabolites are able to activate an estrogen-responsive luciferase reporter gene construct (Meerts et al. 2001).

Our research has shown that DE-71 acts as a weak estrogen in cultured human breast cancer cells and in ovariectomized mice (Mercado-Feliciano M, Bigsby RM, unpublished data). Cell proliferation was increased when MCF-7 cells were cultured in the presence of DE-71 for 10 days. When DE-71 was administered to mice for 34 days, the uterine weight, uterine epithelial height, and vaginal epithelial thickness increased slightly. However, short-term treatment of cultured cells or mice showed very little estrogenic effect. These observations suggest that accumulation of DE-71 metabolites may be involved in these estrogenic effects. Therefore, we conducted the present study to identify and quantify metabolites of DE-71 formed during *in vivo* treatment of mice. Based on these results, three metabolic pathways are proposed.

## Materials and Methods

### Chemicals

We obtained commercial DE-71 from the Great Lakes Chemical Corporation (West Lafayette, IN). Dimethyl sulfoxide and β-estradiol-3-benzoate were purchased from Sigma Chemical Co. (St. Louis, MO), and corn oil was purchased from ICN Biomedicals (Aurora, OH).

We purchased all of the neutral standards (BDEs 28, 47, 71, 77, 85, 99, 100, 153, 154, 183, and 1,3,5-tribromobenzene) from AccuStandard (New Haven, CT). 4′-HO-2,2′,4-Tri-BDE (4′-HO-BDE-17); 2′-HO-2,4,4′-tri-BDE (2′-HO-BDE-28); 4-HO-2,2′,3,4′-tetra-BDE (4-HO-BDE-42); 3-HO-2,2′,4,4′-tetra-BDE (3-HO-BDE-47); 5-HO-2,2′,4,4′-tetra-BDE (5-HO-BDE-47); 6-HO-2,2′,4,4′-tetra-BDE (6-HO-BDE-47); 4′-HO-2,2′,4,5′-tetra-BDE (4′-HO-BDE-49); 2′-HO-2,3′,4,4′-tetra-BDE (2′-HO-BDE-66); and 2′-HO-2,3′,4,5′-tetra-BDE (2′-HO-BDE-68) were gifts from Göran Marsh (Stockholm University, Stockholm, Sweden), and were synthesized as described elsewhere (Marsh et al. 2004). 4-Methoxy-2,2′,3,4′,5-penta-BDE (4-MeO-BDE-90); 5′-methoxy-2,2′,4,4′,5-penta-BDE (5′-MeO-BDE-99); 6′-methoxy-2,2′,4,4′,5-penta-BDE (6′-MeO-BDE-99); 2,3-dibromophenol (2,3-DBP); 2,5-DBP, 3,5-DBP; and 2,4,6-tribromophenol (2,4,6-TBP) were from AccuStandard. 2,4-DBP; 2,6-DBP; 2,4,5-TBP; and 4-HO-2,2′,3,4′,5,5′,6-hepta-BDE (4-HO-PCB-187) were from Cambridge Isotope Laboratories (Cambridge, MA).

All of the phenolic compounds were methylated with fresh diazomethane, which was prepared from Diazald (Sigma Chemical Co.) (Black 1983). All the solvents used for the extraction and cleanup procedures were residue-analysis grade.

### Experimental design

All animal work was approved by the Institutional Animal Care and Use Committee at the Indiana University School of Medicine, and the mice were treated humanely and with regard for alleviation of suffering in the experiment process. Adult BALB/c mice were ovariectomized at 6–8 weeks of age. Starting 3 weeks after the operation, the animals were treated five times per week for 34 days with vehicle or test compound. In brief, groups of five to six animals were treated by either subcutaneous (sc) injection or oral gavage with vehicle control, 10 μg/kg β-estradiol-3-benzoate, or 45 mg/kg DE-71. Some groups were treated with both β-estradiol-3-benzoate and DE-71 at the same time. Chemicals were first dissolved in dimethyl sulfoxide and then diluted in corn oil for a total dose volume of 0.1 mL for oral gavage and 10–20 μL for sc injection. On the day after the last treatment, the animals were sacrificed by decapitation, and blood was collected by exsanguination. Blood was kept at 4°C for ≤ 20 hr before it was centrifuged at 10,000 × *g* for 10 min at 4°C. The supernatant plasma was collected and stored below –20°C until analysis. Because we focused only on the precursor congeners and metabolites of DE-71 in this study, we divided the plasma samples into three groups: oral gavage, sc injection, and controls (plasma samples from mice not treated with DE-71).

### Sample extraction and preparation

A previous method (Hovander et al. 2000; Malmberg et al. 2005) was modified slightly to accommodate the smaller mouse plasma samples in the present study. We transferred each sample (range, 0.028–0.213 g, with an average wet weight of 0.120 g) to a centrifuge tube with a Teflon-lined screw cap. Known amounts of BDE-77 and 4-HO-PCB-187 were added as surrogate standards to determine the recovery of both neutral and phenolic target compounds. After adjustment to 2 mL with 1% potassium chloride, each sample was denatured with 0.5 mL hydrochloric acid (6 M) and 3 mL 2-propanol. Samples were extracted with hexane:methyl *tert*-butyl ether (1:1, vol/vol) three times by shaking the tube > 5 min each time. After blowing down the combined extracts, the phenolic compounds were separated from the neutrals by partitioning with potassium hydroxide (0.5 M in 50% ethanol). The aqueous phase was re-extracted with hexane twice more. After acidification of the aqueous phase, the phenolic compounds were extracted three times with hexane:methyl *tert*-butyl ether (9:1, vol/vol).

The neutral fraction was treated with concentrated sulfuric acid, followed by alumina column chromatography (0.6 cm i.d. × 6 cm, with 0.5 cm anhydrous sodium sulfate on top). The column was eluted with 8 mL hexane followed by 8 mL hexane:dichloromethane (3:2, vol/vol); the PBDE congeners eluted in the second fraction. After concentration, BDE-71 was added as an internal standard for gas chromatographic mass spectrometry (GC/MS) analysis.

The phenolic fraction was concentrated, and the solvent was changed to hexane. To methylate the hydroxyl group, the samples were treated with an excess of a diazomethane solution at room temperature for 3 hr, and the solvent was removed by blowing it down to near dryness. The samples were dissolved in dichloromethane and purified on a column with 0.5 g sulfuric acid–impregnated silica gel, using 8 mL dichloromethane as the mobile phase. After replacement of dichloromethane with hexane, internal standards (1,3,5-tribro-mobenzene for the methylated bromophenols and BDE-71 for the methylated HO-PBDEs) were added for GC/MS analysis.

In each batch of samples, at least two blank samples (phosphate-buffered saline) or control samples (plasma from untreated mice) were prepared. To prevent photodegradation, during the entire process the centrifuge tubes remained wrapped with aluminum foil, or amber vials were used.

### Instrumental analysis

We analyzed both neutral and methylated phenolic fractions using GC/MS [Agilent 6890/5973, with an electron capture negative ionization (ECNI) ion source (Agilent Technologies, Santa Clara, CA)]. We used ECNI-selected ion monitoring of *m/z* 79 and 81 for quantitation. GC injections (1 μL) were made in the pulse splitless mode, with a purge time of 2.0 min. Both the injection port and the transfer line were held at 285°C. A 15-m DB-5-MS capillary column (250 μm i.d., 0.25 μm film thickness; J&W Scientific, Folsom, CA) was used for the neutral fraction, with the following GC oven temperature program: held at 100°C for 1 min; 20°C/min to 200°C; 8°C/min to 270°C; 25°C/min to 300°C; held for 3 min. The same instrument, but with a longer (60-m) column, was used for the methylated phenolic fraction, with the oven temperature program as follows: held at 60°C for 1 min; 3°C/min to 160°C; 15°C/min to 250°C; 2°C/min to 300°C; held for 10 min. We used GC/MS with an electron impact ion source and with a 30-m DB-5-MS column to obtain electron impact mass spectra of target compounds in the full-scan mode.

## Results and Discussion

We detected and quantified all of the main congeners in DE-71 (BDEs 47, 85, 99, 100, 153, and 154) and trace amounts of BDE-28 in the neutral fraction of the mouse plasma samples. No methoxylated PBDEs (MeO-PBDEs) were detected in the neutral fraction. In the phenolic fraction, three bromophenols (2,4-DBP, 2,4,5-TBP, and 2,4,6-TBP) and six HO-PBDEs (4′-HO-BDE-17, 2′-HO-BDE-28, 4-HO-BDE-42, 3-HO-BDE-47, 6-HO-BDE-47, and 4′-HO-BDE-49) were identified and quantified. 4-HO-BDE-90 was identified but not quantified because of co-elution with another peak. Several other potential phenolic metabolites (5-HO-BDE-47, 2′-HO-BDE-68, 6′-HO-BDE-99, 5′-HO-BDE-99, 2,3-DBP, 2,5-DBP, 2,6-DBP, and 3,5-DBP) were not detected. The concentration data for the neutral and phenolic compounds are presented in Table 1.

By using a nonpolar capillary column (such as DB-5) to separate the methylated HO-PBDEs, 2′-HO-BDE-66 will co-elute with 3-HO-BDE-47 (Marsh et al. 2006). However, based on its mass spectrum, the GC peak we observed should be mainly the methyl derivative of 3-HO-BDE-47. Marsh et al. (2006) also reported that the concentration of 2′-HO-BDE-66 was much lower than that of 3-HO-BDE-47 in rat feces after rats were exposed to BDE-47. Thus, in this study we did not determine the concentration of 2′-HO-BDE-66.

The amounts of most target compounds in the blank samples were negligible except for 2,4,6-TBP, for which the blank average concentration was > 50% of that measured in the exposed samples. Relatively high blank concentrations of 2,4,6-TBP are reasonable because this compound is the most common TBP isomer. The recoveries (mean ± SD) of surrogate standards were 96 ± 7% for BDE-77 and 96 ± 5% for 4-HO-PCB-187.

### Neutral compounds

**Figure 1:**
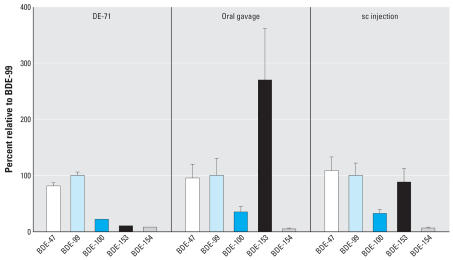
Concentration ratio (%) of neutral congeners normalized to BDE-99 = 100% in DE-71 and mouse plasma samples. Error bars indicate SD.

DE-71 is commercial penta-BDE, and its main components are BDE-47 and BDE-99 in about equal amounts. Some other significant congeners, such as BDEs 100, 153, and 154 (Table 1), are also present in this commercial product. To evaluate the behavior of these congeners in mouse blood, we normalized their concentrations to that of BDE-99 in DE-71 and in mouse plasma as shown in Figure 1.

As shown in Figure 1, the congener ratios of BDEs 47, 100, and 154 are about the same in mouse plasma as in DE-71. However, BDE-153 behaved quite differently. The ratio of BDE-153 to BDE-99 was < 10% in DE-71; however, this ratio increased to 88 ± 24% in plasma samples following sc injection, and it increased to 270 ± 93% after oral gavage. BDEs 47, 99, and 153 represented 18, 19, and 52%, respectively, of total measurable PBDEs in oral gavage samples, whereas they represented 36, 44, and 4.3% of the total in the DE-71 mixture (Table 1). The increased ratio of BDE-153 suggests that this compound was poorly metabolized or otherwise highly persistent in mice.

In orally exposed mice, two factors might influence the concentration of PBDEs in blood: uptake efficiency and removal rate. Burreau et al. (1997) reported uptake efficiencies of 90, 60, and 40% for BDEs 47, 99, and 153, respectively, in pike (*Esox lucius*) fed a mixture of these compounds. Other researchers also showed that BDE-153 was less absorbed than BDE-47 and BDE-99 in rats and mice following gavage (Chen et al. 2006; Sanders et al. 2006a, 2006b). The uptake efficiency for BDE-153 was lower than that of BDE-47 and BDE-99, probably because of its larger size.

Two processes are able to remove PBDEs from blood: distribution between blood and other tissue (mainly adipose tissue) and metabolism. Because adipose tissue is the main tissue sink of BDE-153 in mice (Staskal et al. 2006) and because others have found either similar BDE-99 and BDE-153 distributions to adipose tissue (Sanders et al. 2006b) or higher adipose tissue distribution for BDE-153 than for BDE-99 or BDE-47 (Staskal et al. 2006) in exposed rodents, it seems that sequestration to tissues cannot explain the higher blood concentration of BDE-153 compared to BDE-99. Furthermore, both uptake and distribution are biophysical processes, which are likely to have little isomer selectivity. For example, BDE-154 and BDE-153 should have almost the same absorption and distribution behavior because these two isomers are close in structure and in their physicochemical properties, especially their *K*_ow_ values (Braekevelt et al. 2003). We found no relative increase of BDE-154 in mouse plasma samples compared with its relative concentration in DE-71 (Table 1, Figure 1); therefore, differential metabolism is the more likely explanation for the accumulation of BDE-153 in mice.

Metabolic enzymes, which might have high isomer selectivity, could determine the concentration pattern of congeners in tissues, as well as their fate and potential toxicity (Hakk and Letcher 2003). In mammals, the liver is the main site where xenobiotic chemicals are metabolized. High concentrations of BDE-153 in blood might suggest that BDE-153 was resistant to enzyme degradation in mouse liver. Sanders et al. (2006b) reported that when rats were exposed to a mixture of BDE-47, BDE-99, and BDE-153, the concentration of BDE-153 in liver was three times higher than that of BDE-47 and BDE-99.

BDE-153 was only a minor component in both commercial penta-BDE and octa-BDE technical mixtures (Birnbaum and Staskal 2004). In field samples, such as sediment and ambient and indoor air, no obvious evidence of BDE-153 accumulation was found when compared to BDE-47 and BDE-99 (Hites 2004). This is expected for congeners that have comparable environmental fates; that is, they have similar physicochemical pathways from their sources to the environmental media from which they are sampled. Conversely, relatively high concentrations of BDE-153 have been measured in samples from some wild animals and humans, especially in liver and blood (plasma or serum) samples. Voorspoels et al. (2006) reported that, in the liver of red foxes from Belgium, the concentration of BDE-153 was > 10 times higher than that of BDE-99 and 3 times higher than that of BDE-47. Recently, relatively high concentrations of BDE-153 were also observed in human sera, especially from the Netherlands and Sweden (Weiss et al. 2004, 2006).

Although it has been proposed that debromination of BDE-209 may be a source of BDE-153 (Weiss et al. 2006), at present there is no evidence to support this hypothesis. When carp were exposed to BDE-209, the congeners BDE-154, BDE-155, and another unidentified hexa-BDE were the three main hexabrominated products (Stapleton et al. 2006a), suggesting that BDE-153 was not preferentially formed from BDE-209, at least in fish. In the present study, BDE-209 was not present to serve as a precursor of BDE-153. Therefore, resistance to enzyme degradation is likely to be the most important cause of the increased relative concentration of BDE-153 observed in various animals.

### Phenolic compounds

HO-PBDEs are metabolites of PBDEs in animals, and they can also occur in marine environments as natural products (Malmvärn et al. 2005a). These compounds have been observed in animals from *in vivo* experiments (Malmberg et al. 2005; Marsh et al. 2006) and in field samples (sometime in the form of methylated derivatives) (Malmvärn et al. 2005a, 2005b; Marsh et al. 2004; Stapleton et al. 2006b; Valters et al. 2005). In the present study, we identified many phenolic metabolites, limited only by the availability of standards. Based on the metabolites we measured, there seem to be three main metabolic pathways for PBDEs in mice: cleavage of the diphenyl ether bond, hydroxylation, and debromination/hydroxylation.

Table 1 lists the phenolic compounds measured in the mouse plasma samples, including their concentrations in animals dosed by oral gavage and by sc injection. All of these metabolites were identified not only by comparing their retention times to those of authentic standards but also from their mass spectra, which were obtained using GC/MS with an electron impact ion source operating in full-scan mode.

#### Cleavage of the diphenyl ether bond

Most recent research has focused on the hydroxylated PBDE metabolites, but few articles have reported diphenyl ether bond cleavage. Omitting 2,4,6-TBP because of blank interferences, we have identified two bromophenol metabolites (2,4-DBP and 2,4,5-TBP), which could only be obtained through the cleavage of the diphenyl ether bond. These bromophenols were detected at concentrations higher than most of the two-ring hydroxylation products (Table 1), suggesting that diphenyl ether bond cleavage is an important metabolic pathway for PBDEs in mice.

**Figure 2:**
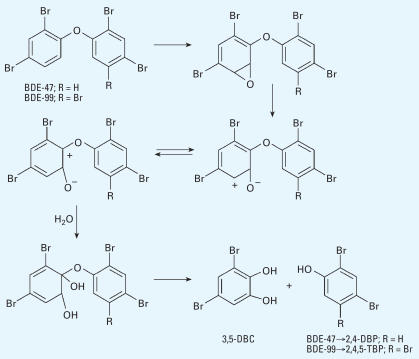
Proposed pathway of the cleavage of the diphenyl ether bond of BDE-47 and BDE-99 in mice.

Previous studies have reported the cleavage of the diphenyl ether bond of PBDEs in the gas phase (Raff and Hites 2006) and of a PBDE-like thyropropionic acid in solution (Matsuura et al. 1971). The mechanism proposed for such reactions involves a HO radical attacking the carbon–oxygen bond. A similar mechanism has been reported to cleave the thyroxine diphenyl ether bond *in vitro* by incubation with rat liver homogenates (Balsam et al. 1983). In the case of PBDEs, the intermediate product then cleaves into a brominated phenol and a phenoxy radical, which can abstract hydrogen to form another brominated phenol. In this potential pathway, 2,4,5-TBP could come mainly from BDE-99, whereas 2,4-DBP could come from both BDE-47 and BDE-99; therefore, the concentration of 2,4-DBP would be expected to be greater than that of 2,4,5-TBP. However, our measurements showed a concentration ratio of 2,4-DBP to 2,4,5-TBP of 0.89 (the molecular ratio was 1.2), which is near the concentration ratio of BDE-47 to BDE-99 in DE-71 (0.82; the molecular ratio was 0.96). Although the ratio of 2,4-DBP to 2,4,5-TBP might be influenced by different residence times of these two compounds in blood, this ratio could also suggest the occurrence of another metabolic step in animals that does not occur in the gas phase or in solution. For example, BDE-99 could produce one molecule of 2,4,5-TBP and one molecule of 3,5-dibromocatechol (3,5-DBC) through an arene oxide intermediate (Chen et al. 2006), and BDE-47 could produce 2,4-DBP and 3,5-DBC, as shown in Figure 2.

The same degradation pathway was proposed by Schmidt et al. (1993) when halogenated diphenyl ethers were degraded by the bacterium *Sphingomonas* sp. Strain SS3. In fact, the bacterial scission of ether bonds is an important degradation pathway for compounds with ether linkages in the environment, including diphenyl ethers (White et al. 1996). We suggest that this pathway is important not only in bacterial degradation but also in mammals. However, at present there are no reports in the literature of bromocatechol as a PBDE metabolite in rodents or in other animals. Because there was no standard available for 3,5-DBC, we did not measure this compound. Identification of 3,5-DBC as an animal metabolite in future studies will help further our understanding of the cleavage of the diphenyl ether bond in PBDEs.

#### Hydroxylation

**Figure 3:**
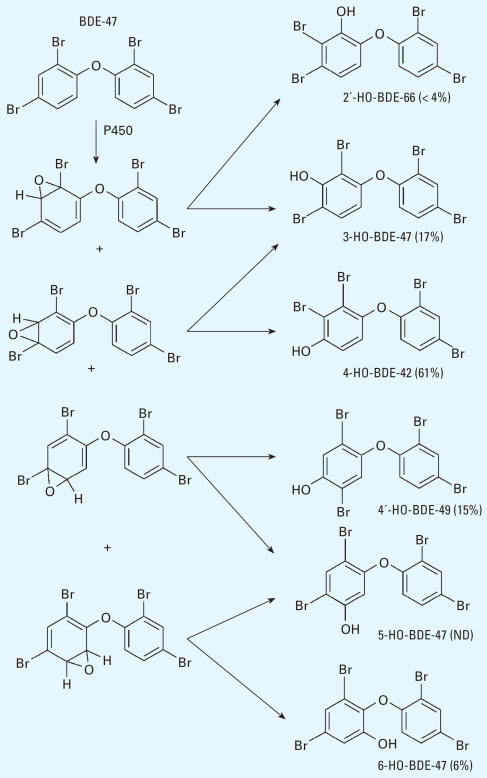
Proposed hydroxylation pathway of BDE-47 in mice and the percentage of metabolites based on the measurements in this study. ND, not detected.

Hydroxylation is an important metabolic pathway for PBDEs in animals. We identified and quantitated almost all possible HO-tetra-BDE peaks in mouse plasma (Table 1). If we assume that all HO-tetra-PBDEs came from arene oxide intermediates of BDE-47 and that all of these HO-PBDEs have the same residence time in mouse blood, then we can propose a metabolic pathway and estimate the product proportions as shown in Figure 3.

**Figure 4:**
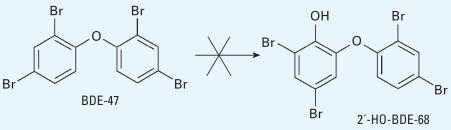
BDE-47 cannot be metabolized to 2′-HO-BDE-68.

Another potential metabolite, 2′-HO-BDE-68, was not detected, suggesting that there was no possible way to form this compound from BDE-47 (Figure 4).

We suspect that hydroxylation of PBDEs is mediated by cytochrome P450 enzymes, which can account for direct hydroxylation or hydroxylation via a 1,2-shift. The 1,2-shift mechanism proceeds via an arene oxide intermediate (Guroff et al. 1967; Jerina and Daly 1974). Obviously, 4-HO-BDE-42 and 4′-HO-BDE-49 must have been formed through a 1,2-shift. The methylated products of 4-HO-BDE-42 and 4′-HO-BDE-49 have also been measured in field samples (Valters et al. 2005). It is notable that these *para*-HO-PBDEs in our samples totaled > 75% of the total measurable HO-tetra-BDE metabolites. This suggests that when the hydroxylation of BDE-47 occurred, the HO group was more likely to transfer to the *para* position of the phenyl ring with a 1,2-shift of the original *para-*bromine atom. 6-HO-BDE-47 was present at a low abundance (6%), and 5-HO-BDE-47 was not detected. Overall, our results suggest that hydroxylation has a preference for the *para* position as opposed to the *ortho* or *meta* positions.

The high proportion of some HO-PBDEs, such as 4-HO-BDE-42, suggests that either they were preferentially formed or were more persistent in mouse blood. These metabolites might have a substantial biological effect. For example, three HO-PBDE congeners (4-HO-BDE-42, 4′-HO-BDE-49, and 3-HO-BDE-47) were shown to have up to a four times stronger affinity to TTR than does thyroxine (Malmberg et al. 2005). This suggested that *para-*HO-PBDEs might be the most thyroxine-like HO-PBDEs. In any case, the preferential formation and/or persistence of *para-*HO-PBDEs in mice suggests these compounds should receive more toxicologic attention.

**Figure 5:**
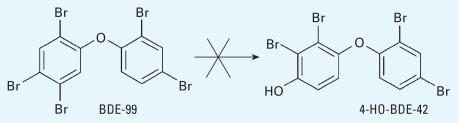
BDE-99 cannot be metabolized to 4-HO-BDE-42.

As discussed above, we assumed that HO-tetra-BDEs result from the hydroxylation of BDE-47. HO-PBDEs could also result from debromination/hydroxylation reactions of BDE-99, but these reactions may be less important than hydroxylation alone. Moreover, by comparing the structure of HO-tetra-BDEs, such as 4-HO-BDE-42, with BDE-47 and BDE-99, it is apparent that these HO-tetra-BDEs more likely come from hydroxylation of BDE-47 rather than from debromination/hydroxylation of BDE-99 (Figure 5).

In addition to the mono-hydroxylated tetra-BDEs, one dihydroxylated tetra-BDE was identified through its mass spectrum, but we were unable to determine its substitution pattern. Others have made a similar observation (Malmberg et al. 2005).

**Figure 6:**
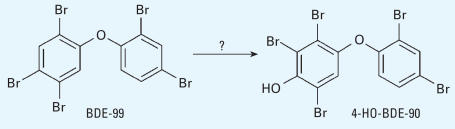
Speculation on the metabolism of BDE-99 to 4-HO-BDE-90.

For the three HO-*penta-*BDE isomers for which we had standards (5′-HO-BDE-99, 6′-HO-BDE-99, and 4-HO-BDE-90), only 4-HO-BDE-90 was detected, but we found two other relatively abundant GC peaks corresponding to other HO-*penta*-BDEs, which together could represent the most abundant HO-*penta-*BDEs (Figure 6).

The electron impact mass spectra of these two peaks indicated that the hydroxyl group was in the *meta-*position of the phenyl ring. We observed ions due to [M]^+^, [M–2Br]^+^, and [M–2Br–CH_3_]^+^, which were consistent with a methylated derivative of a *meta*-HO-BDE structure as described by Malmberg et al. (2005). The preferential formation of *meta-*HO-BDEs from a *penta-*BDE, presumably BDE-99, was quite different from the hydroxylation of BDE-47, for which *para-*HO-BDEs were the major metabolites.

#### Debromination/hydroxylation

**Figure 7:**
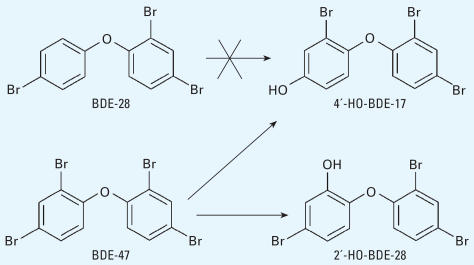
Debromination/hydroxylation of BDE-47 can give two hydroxylated tri-BDEs, but BDE-28 cannot.

The metabolic pathway of debromination/hydroxylation has also been observed in other studies. When rats were exposed to BDE-47, three kinds of HO-tri-BDE were found in feces (Marsh et al. 2006). In the present study, we found and quantitated two HO-tri-BDEs (4′-HO-BDE-17 and 2′-HO-BDE-28). Although we found trace amounts of BDE-28 in commercial DE-71 and it was detected in mouse plasma, the concentration of BDE-28 was much lower than those of 4′-HO-BDE-17 and 2′-HO-BDE-28 (Table 1). It is more reasonable to believe that these two isomers were formed by the debromination/hydroxylation of a more abundant congener, such as BDE-47, rather than by hydroxylation of BDE-28 (Figure 7).

Moreover, although there might be a 1,2-shift during the hydroxylation process and bromine could shift to the adjacent position on the phenyl ring, at present there is no indication that bromine can shift from a *para* position to an *ortho* position. In other words, 4′-HO-BDE-17 could not be formed from BDE-28, leaving debromination/hydroxylation of a more brominated BDE (such as BDE-47) as the more likely pathway (Figure 7).

Compared with those metabolites formed through direct hydroxylation, the concentration of metabolites formed by debromination/hydroxylation was low (Table 1), which suggests that hydroxylation is a more important process than debromination/hydroxylation, at least for tetra-BDE.

## Conclusion

BDE-153 accumulates in the blood of mice exposed to commercial DE-71, suggesting that this compound might be resistant to enzymatic degradation. For the other PBDE congeners in DE-71, there seem to be three metabolic pathways: cleavage of the diphenyl ether bond, hydroxylation, and debromination/hydroxylation. The cleavage of the diphenyl ether bond forms bromophenols, whereas the other two pathways form HO-PBDEs, in some cases with a 1,2-shift of the original *para-*bromine atom. In this pathway, *para-*HO-PBDEs are most likely formed from BDE-47; these metabolites could be the most thyroxine-like and/or estrogen-like congeners among the HO-PBDEs.

## Correction

In Figure 3 of the original manuscript published online, the arrows indicating pathways were incorrect. They have been corrected here.

## Figures and Tables

**Table 1:** Concentrations (ng/g wet weight) of neutral and phenolic compounds in mouse plasma.

	Oral gavage samples (*n* = 15)		sc injection samples (*n* = 14)		Blank and control samples (*n* = 19)
Compound/congener	Mean ± SD	Percent of total	Mean ± SD	Percent of total	Mean ± SD
Neutral compounds^a^					
BDE-28 (0.3)	4.4 ± 1.1	0.2	5.3 ± 1.6	0.5	0.6 ± 0.8
BDE-47 (36)	390 ± 100	18	360 ± 77	31	3.0 ± 6.3
BDE-85 (2.6)	57 ± 19	2.6	32 ± 5	2.8	ND
BDE-99 (44)	410 ± 120	19	330 ± 70	29	2.3 ± 3.9
BDE-100 (9.1)	140 ± 40	6.4	110 ± 20	9.2	0.4 ± 1.3
BDE-153 (4.3)	1,100 ± 380	52	290 ± 80	25	0.6 ± 0.8
BDE-154 (3.3)	22 ± 7	1.0	20 ± 4	1.7	0.1 ± 0.2
Total	2,150 ± 410	100	1,150 ± 130	100	
Phenolic compounds					
2,4-DBP	72 ± 23	15	62 ± 25	17	1.4 ± 3.8
2,4,5-TBP	79 ± 29	16	86 ± 40	24	0.3 ± 0.6
2,4,6-TBP	5.3 ± 3.4	1.1	6.0 ± 6.0	1.6	3.3 ± 3.2
4′-HO-BDE-17	17 ± 10	3.5	11 ± 7	3.0	ND
2′-HO-BDE-28	11 ± 6	2.3	5.2 ± 2.5	1.4	0.1 ± 0.2
4-HO-BDE-42	180 ± 120	38	120 ± 88	32	1.1 ± 2.7
3-HO-BDE-47	53 ± 25	11	33 ± 17	9.1	ND
6-HO-BDE-47	22 ± 12	4.6	8.5 ± 4.0	2.3	ND
4′-HO-BDE-49	42 ± 22	8.7	34 ± 19	9.3	0.3 ± 0.8
Total	480 ± 130	100	360 ± 104	100	

ND, not detected.

a Congeners (percentages) found in DE-71.
